# Modelling the COVID-19 pandemic in context: an international participatory approach

**DOI:** 10.1136/bmjgh-2020-003126

**Published:** 2020-12-23

**Authors:** Ricardo Aguas, Lisa White, Nathaniel Hupert, Rima Shretta, Wirichada Pan-Ngum, Olivier Celhay, Ainura Moldokmatova, Fatima Arifi, Ali Mirzazadeh, Hamid Sharifi, Keyrellous Adib, Mohammad Nadir Sahak, Caroline Franco, Renato Coutinho, CoMo Consortium, Proochista Ariana

**Affiliations:** 1 Nuffield Department of Medicine, University of Oxford Centre for Tropical Medicine and Global Health, Oxford, Oxfordshire, UK; 2 MAEMOD, Mahidol Oxford Tropical Medicine Research Unit, Bangkok, Thailand; 3 Center for Tropical Medicine and Global Health, University of Oxford Centre for Tropical Medicine, Oxford, UK; 4 Weill Cornell Medicine, Cornell Institute for Disease and Disaster Preparedness, New York, New York, USA; 5 Nuffield Department of Medicine, University of Oxford, Oxford, Oxfordshire, UK; 6 Department of Epidemiology, Florida International University, Miami, Florida, USA; 7 School of Medicine, University of California San Francisco, San Francisco, California, USA; 8 WHO Collaborating Center for HIV Surveillance, Institute for Futures Studies in Health, Kerman University of Medical Sciences, Kerman, Iran, Kerman, Iran (the Islamic Republic of); 9 Independent Researcher (No affiliation), no affiliation, UK; 10 Regional Office for the Eastern Mediterranean, World Health Organization, Kabul, Afghanistan; 11 Waves and Non-Linear Patterns Research Group, São Paulo State University (UNESP), Institute of Theoretical Physics, Sâo Paulo, Sao Paulo, Brazil; 12 Centre for Mathematics, Computation and Cognition, Federal University of ABC Center of Mathematics Computing and Cognition, Santo Andre, São Paulo, Brazil

**Keywords:** health policy, respiratory infections, control strategies, SARS

## Abstract

The SARS-CoV-2 pandemic has had an unprecedented impact on multiple levels of society. Not only has the pandemic completely overwhelmed some health systems but it has also changed how scientific evidence is shared and increased the pace at which such evidence is published and consumed, by scientists, policymakers and the wider public. More significantly, the pandemic has created tremendous challenges for decision-makers, who have had to implement highly disruptive containment measures with very little empirical scientific evidence to support their decision-making process. Given this lack of data, predictive mathematical models have played an increasingly prominent role. In high-income countries, there is a long-standing history of established research groups advising policymakers, whereas a general lack of translational capacity has meant that mathematical models frequently remain inaccessible to policymakers in low-income and middle-income countries. Here, we describe a participatory approach to modelling that aims to circumvent this gap. Our approach involved the creation of an international group of infectious disease modellers and other public health experts, which culminated in the establishment of the COVID-19 Modelling (CoMo) Consortium. Here, we describe how the consortium was formed, the way it functions, the mathematical model used and, crucially, the high degree of engagement fostered between CoMo Consortium members and their respective local policymakers and ministries of health.

**Key questions**

**What is already known?**

- The optimal approaches to tackle the COVID-19 pandemic depend on several contextual factors, including population age structure, variations in available resources (including infrastructure, financial and human resources) and sociocultural considerations.

- Governments across the world have been advised by mathematical modelling projections that do not necessarily take those contextual factors into consideration.

**What are the new findings?**

- We describe the creation of a participatory modelling approach platform, the COVID-19 Modelling Consortium, and illustrate some of its use cases.
- We demonstrate how the participatory nature of the consortium has been critical in its success, in terms of addresing the contextual factors that underpin health policy interventions and gaining decision makers' trust.

**What do the new findings imply?**

- We advocate a participatory modelling approach, where in-country experts play an essential iterative role, being policy-facing in its dealings with policymakers and simultaneously delivering or facilitating reactive modelling that can feed back, in real time, into the decision-making processes.

## Introduction

The novel coronavirus, SARS-CoV-2, which causes COVID-19, has affected at least 213 countries/regions, with more than 17 million confirmed cases and in excess of 660 000 deaths globally.^1^ As a new clinical entity, the global impact of COVID-19 is characterised by both uncertainty and rapid discovery, laying the grounds for mathematical modelling to emerge as the prominent field of research used to provide advice for pandemic containment strategies.^2 3^ High-income Asian countries were able to call on their system responsiveness and experience with recent pandemics, rapidly enforcing efficient testing and quarantining/isolation strategies. In contrast, European countries took a much more measured approach (easily confused with lack of preparedness) early on, tapping into their modelling expertise to predict the outcome of the pandemic and what the best containment strategies moving forward might be. As a result, most European countries converged and introduced suppression strategies (including everything from the shutdown of basic economic activity, in many instances enforced by hastily promulgated laws or executive orders, to the wholesale reorganisation of medical and hospital-based care) centred around non-pharmaceutical interventions (NPIs),^4–6^ fearing that pandemic mitigation would cost too many lives, with the notable exception of Sweden.^7^ Interestingly, several low-income and middle-income countries (LMICs) followed suit, adopting health policies informed by early modelling from developed countries, without considering how the modelling predictions might be affected by contextual factors. Modelling devoid of local context has been shown to produce far from optimal/useful projections in the Ebola, H5N1 and H1N1 pandemics/outbreaks.^2 8^ For such high priority and complex topics, a participatory modelling approach seems to be particularly well suited, as it provides policymakers with timely and dynamic support, enabling the modelling process to be built around the coproduction of knowledge between modellers and policymakers.^9^ This multipronged approach with collaboration among experts of different disciplines working together to incorporate all relevant contextual factors into pandemic modelling has been discussed at length in Rhodes *et al*.^8^ Here, we describe a participatory approach to modelling that coalesced around these contextual considerations and resulted in the creation of an international consortium of infectious disease modellers and other public health experts, the COVID-19 Modelling (CoMo) Consortium. Putting in-country experts at the forefront of model development, we underscore the need to incorporate contextual factors (including population age structure, resource availability—including infrastructure, financial and human resources—and sociocultural considerations) into an iterative policy informing tool. Our approach demands a social–ecological component comprising a combination of the contextual factors listed below as an integral part of the epidemiological model, thus addressing some of the limitations observed in other modelling exercises.^2 3 8 10 11^ We appreciate a compromise between accuracy, transparency, flexibility and timeliness remains, but are confident a participatory approach is the best avenue to minimise those trade-offs.

## Contextual factors

### Contextual factor 1: population age structure

It became clear during the early stages of the COVID-19 pandemic that a disproportionate number of older individuals are at higher risk of severe disease and mortality,^12 13^ with 80% of deaths associated with the disease occurring in those aged more than 65 years in outbreaks in China and the USA.^13 14^ In Italy, the proportion of deaths occurring in people over the age of 70 reached 88%, presumably due to an aged population in the Lombardy region.^15^ Data from the Chinese and Italian outbreaks suggest that the case fatality rate (CFR) was <1% in the under-50s, rising to almost 15% and 20% in the over-80s, respectively.^15 16^ Children appear to suffer from less severe symptoms^17^ but are at a similar risk of being infected as the general population; therefore, the role played by children in the transmission of COVID-19 should be considered when developing control strategies.^18^ This variation in the age-dependent severity of COVID-19 has important implications for the impact of the disease in any given country.

### Contextual factor 2: uncertainties around the characteristics of the disease

As a newly emerged disease, considerable uncertainty remains around some of the basic parameters of COVID-19 infection. Evidence suggests that the median incubation period is approximately 5 (95% CI: 4.5 to 5.8) days.^19^ The duration of the infectious period is extremely uncertain, with some studies suggesting that people become infectious before developing symptoms^20–22^ and others finding that viral shedding in clinical cases can persist for more than 20 days.^12 23^ In fact, some evidence suggests infectiousness begins before symptoms develop and is likely to peak around the time of symptom onset.^23–25^ Several estimates for the serial interval (time between transmission chains),^26 27^ taken together with estimates for the incubation period, strongly suggest that asymptomatically infected people can transmit the virus. A study from China found the median duration from first symptoms to dyspnoea, hospital admission and acute respiratory distress syndrome was 5, 7 and 8 days, respectively,^28^ although data from New York City suggest a more rapid progression.^29^ The infection fatality rate (IFR) measures the percentage of infected individuals who later succumb to the disease; a meta-analysis suggests an IFR for COVID-19 of around 0.20% (mean across all ages), given all available data as of 22 March 2020.^16^ However, these estimates are full of uncertainties particularly to what concerns the denominator due to challenges in reliably ascertaining how many people are/have been infected with the virus. Two streams of scientific research are trying to resolve this underlying burden of infection: one relies on the use of inference and predictive models,^30–32^ making the best use of available data to disentangle the unobserved number of infections driving the force of infection; the other focuses on diagnostic tool development to enable reliable mass serological studies to be carried out.^33–35^

Reported symptomatic CFR vary widely by country, as criteria and capacity for testing can vary considerably. Burdens of comorbidity differ, as do demographics, and cause of death attribution is not uniform. Both IFR and CFR values may also be dynamic in a single setting, as delays in deaths tend to result in the underestimation of CFR early on in an epidemic, with surges in lethality during healthcare system stress.

Those at higher risk of developing severe disease include individuals with comorbidities such as hypertension, cardiovascular disease and diabetes.^36^ Obesity, especially in younger patients, is emerging as a risk factor for more severe clinical manifestations in cohorts in the USA.^29^ There is conflicting evidence regarding the contribution of pre-existing respiratory disease to clinical severity.^37^ Given the relative paucity of detailed and accurate parameter data, it is vital that local modellers who have intimate knowledge of their own context(s) are engaged to ensure the optimal application and communication of any model and its outputs.

### Contextual factor 3: differences in health system capacity

The COVID-19 pandemic presents myriad challenges for healthcare systems around the world,^38^ including the need to repurpose and train healthcare staff, increase the number of both general hospital beds and intensive care unit beds, purchase equipment (particularly ventilators, high-flow oxygen systems and oxygen concentrators), and hire carers needed for specialist care and/or treatment for comorbidities.^39^ Anticipating health system demand in comparison to capacity under various intervention scenarios is a key aspect of national and regional strategic decision-making.^40^ This underscores one of the key roles of the disease-modelling approach in the context of COVID-19 pandemic preparedness and response.

### Contextual factor 4: socioeconomic and cultural differences

The connection between cultural values (uncertainty avoidance, power distance, individualism vs collectivism) and infectious diseases is well established, primarily with literature on antibiotic prescribing and treatment seeking behaviours.^41^ Different countries have adopted a variety of strategies and combinations of NPIs to meet the challenges posed by COVID-19. While these differences in approach are highly dependent on local cultural contexts and values,^42^ their differential effect in the setting of COVID-19 is debated among social scientists.^43^

Early empirical work is emerging suggesting disease spread was slower in countries with strong institutional systems and hierarchical cultures.^44^ In China, where this novel coronavirus first emerged, a strict policy including measures such as quarantine, self-isolation and containment immediately implemented.^17^ This ultimately involved strict physical distancing measures in social settings, or a ‘lockdown’, as Chinese local authorities imposed travel restrictions and severely restricted the movement of people. Physical distancing and isolation measures, aided by extensive testing and contact tracing, were also used effectively in South Korea to bring their COVID-19 epidemic under control.^45^ Singapore undertook intensive surveillance and contact tracing, followed by isolation of suspected and confirmed cases to halt transmission chains.^35^ Some Muslim countries in the Middle East have made a historical decision to cancel Friday and congregational prayers and to close their holy shrines.^46^ Other countries, such as the UK, started pursuing strategies aimed at allowing herd immunity to gradually develop while reducing the demand on the health service, also known as ‘flattening the curve’, but did eventually enforce a strict lockdown policy. Notably, in Northern Europe, the onus of practicing efficient containment measures was transferred to the individual, while more relaxed lockdown versions were enforced, characterised by a minimal disruption to society. Several recent studies have tried to estimate the impact of these different containment/suppression strategies in different countries,^4 47–52^ but little insight has been gained into the optimal long term strategy, assuming that a vaccine may not be available before the end of the year.

Socioeconomic differences can critically underpin the potential adherence of any infection containment measure. A significant proportion of the population in LMICs are daily-wage earners who cannot work from home. They often rely on street vendors or local markets for their meals, which are usually overcrowded places where hygiene and physical distancing measures are difficult to enforce. Large and intergenerational families, migrants and refugees who live in densely populated areas mean physical distancing is virtually impossible, ineffective and may cause more harm than good. Religious and cultural festivals can seriously disrupt physical distancing measures usually people would gather in the thousands to celebrate Easter, Chinese New Year, Ramadan.

### Contextual factor 5: a rapidly developing situation

Published models frequently remain inaccessible to policymakers in LMICs due to the lack of translational capacity in many of these countries. This can present difficulties in converting a prepublication or peer-reviewed model into a practical, real-time decision-making tool. Critically, any model requires continuous updating, usually on a daily basis, if it is to keep pace with the situation unfolding in a given country and the science relating to the infectious agent it is modelling, and therefore meet the needs of policymakers in that country. Publishing scientific papers and online tools alone is insufficient to engage with and inform health policymakers, because in a rapidly developing situation, the model and the modeller cannot be separated, and policy responses must be couched within the specific country or subnational context. This can only be achieved via a combination of technology, training and communication. User-friendly platforms facilitating real-time data analysis and scenario prediction by local epidemiologists is key for effective planning and policy decision making to mitigate the COVID-19 pandemic.

## The COVID-19 Modeling (CoMo) Consortium

### CoMo Consortium—policy rationale

**Figure 1:**
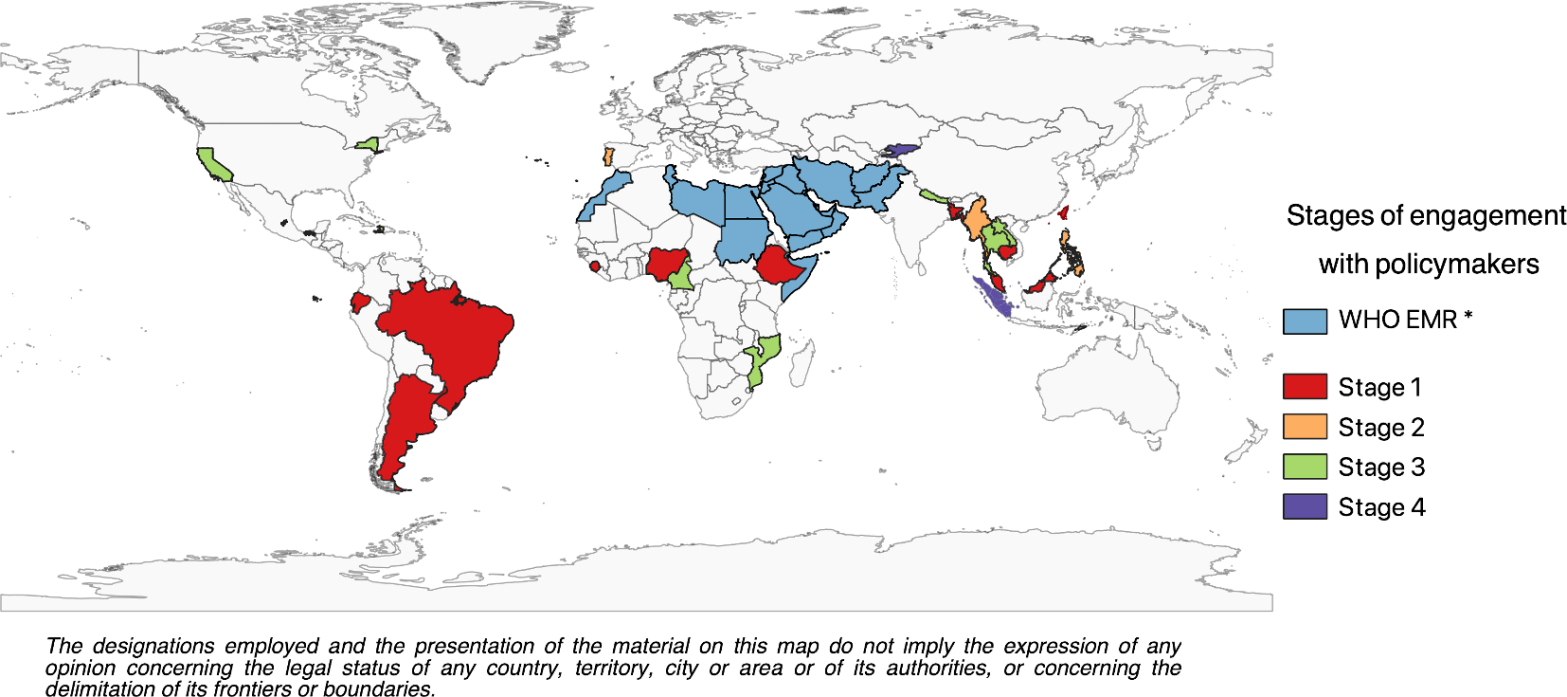
CoMo Consortium participants. Individual country participants, colour coded by the stages of engagement with policymakers (table 2). *Refers to the 22 countries/territories using the CoMo model through the WHO Regional Office for the Eastern Mediterranean (EMRO). The designations employed and the presentation of the material on this map do not imply the expression of any opinion concerning the legal status of any country, territory, city or area or of its authorities or concerning the delimination of its frontiers or boundaries. CoMo, COVID-19 Modelling.

During the acute phase of the pandemic, the focus of most CoMo Consortium modelling work has been to address immediate questions driven by policymakers (figure 1). These have focused on exploring optimal strategies to achieve specific aims, such as minimising cases, mortality or demand on the health system. As the situation evolves, more thought is now being directed towards economic impacts and the direct and indirect costs of the disease and the measures taken to control its spread. The challenges of increasing and repurposing health system capacity, in addition to worker absenteeism due to COVID-19 morbidity and mortality, represent some of the direct costs associated with the pandemic.^53^ The economic and behavioural responses adopted to reduce SARS-CoV-2 transmission form a substantial proportion of the indirect costs associated with the pandemic. These include suspension of manufacturing and production; employment losses and reductions in consumer spending in many sectors, notably travel and tourism;^53^ interruptions in services and the supply chain of products for the prevention and control of other major diseases, such as vaccine-preventable diseases, HIV, malaria and active cancers. A fully comprehensive set of cost efficiency/effectiveness analyses will be of great value to inform countries seeking scientific evidence evaluating current and future containment strategies.

### CoMo Consortium—a participatory approach

Historically, modelling expertise has been concentrated in high-income countries (HICs), where multiple modelling groups, often with large teams, have tended to form consortia. While these consortia mainly inform policy in HICs, they often act as external service providers to LMICs. Modelling inputs to LMICs have, therefore, generally involved two groups of professionals: modellers from HICs and policymakers from LMICs; a situation which is far from ideal. We sought to avoid this by adopting a participatory approach when establishing the international CoMo Consortium. A participatory approach is key for policymakers to fully appreciate the uncertainties subjacent to assumed parameter values, implemented mechanisms of action and general model structure. The immediate consequence of that understanding is clarity on the relevance of critical data in circumventing uncertainties, and the understanding that continual validation frameworks are key to guarantee the best possible policy is implemented at all times. The use of a participatory approach can even be viewed as part of the intervention package, as argued in other studies,^3^ and is highly desirable,^54^ as evidenced by the number of policymakers from multiple countries around the world who have requested to actively participate in the CoMo Consortium. Being a Consortium member also facilitates information sharing among countries with comparable contexts that might be addressing similar questions.

**Figure 2:**
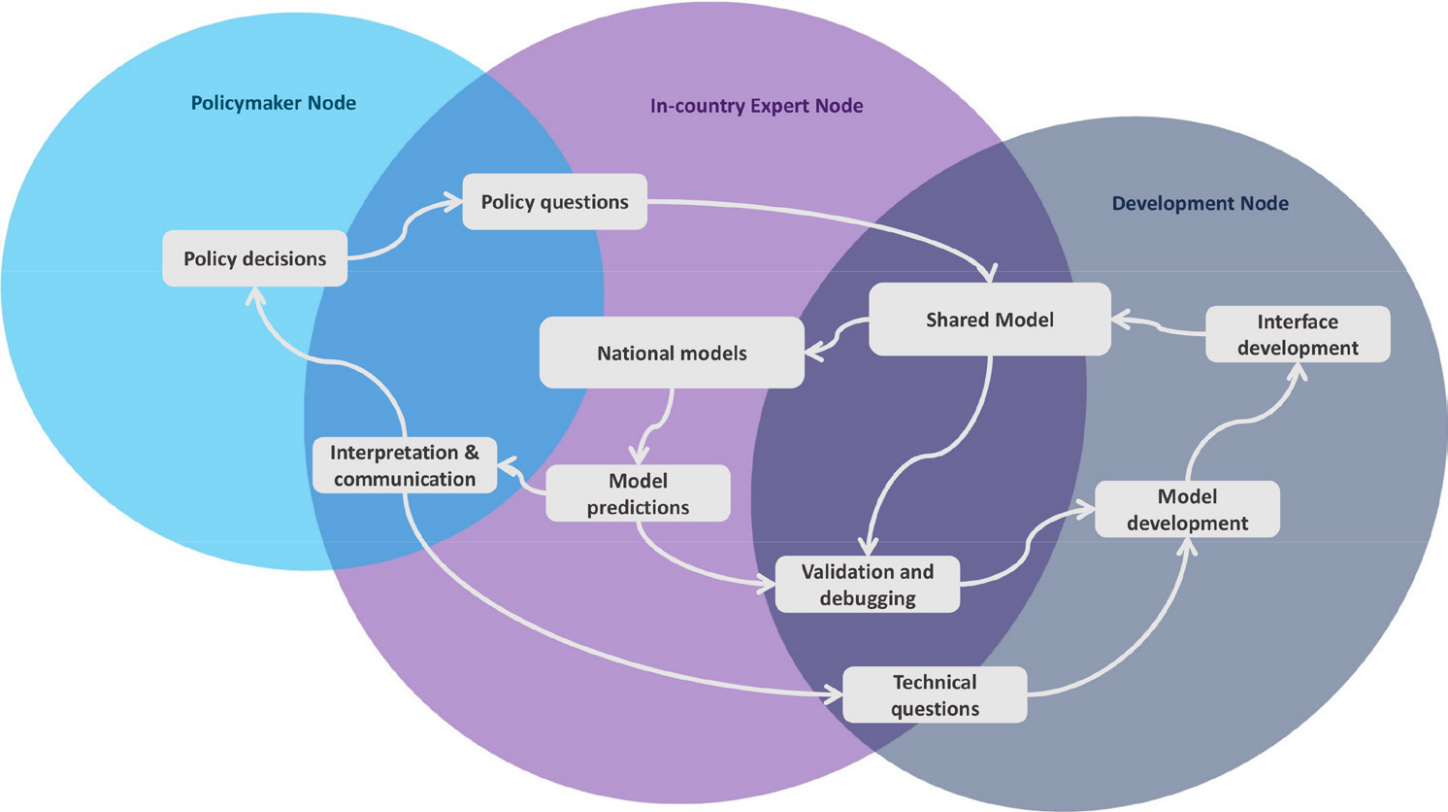
CoMo Consortium outlook and interaction flows. This diagram illustrates how the different partners interact in order to digest policy questions into model simulations through the in-country expert node and the development node and ultimately result in actionable predictions informing policy decisions. CoMo, COVID-19 Modelling.

The CoMo Consortium mathematical model was developed by three groups of professionals, with each group forming one of three nodes: a development node, an in-country expert node and a policymaker node. Each node comprises a variety of relevant professionals (table 1, figure 2). Where there were existing in-country experts, the CoMo Consortium sought to build on existing close working relationships (or establish such relationships) with these experts.

**Table 1:** The three nodes of the CoMo Consortium Development phase

Development node	In-country expert nodes	Policymaker nodes
Lead modeller Experienced modellers Clinician modeller (public health specialist) App developer Coordinator (junior modeller) Economist	Research clinician/preparedness modellers Epidemiologists/surveillance specialists (field/public health) Modellers (early stage/senior) Public health specialists Health economics modellers Medical statisticians Representatives from WHO/other NGOs	State government State ministries of public health National ministries of public health Local governing bodies and their health departments

CoMo, COVID-19 Modelling.

### CoMo Consortium—development phase

The CoMo Consortium was established as a response to the analytical demands of LMICs trying to prepare for the COVID-19 epidemic. Several in-country experts were approached for support by their own policymakers and later reached out to the Oxford Modelling for Global Health group for additional technical advice and support. This sparked an initiative to provide technical support and mentoring to these individuals which evolved into a precursor for the CoMo consortium. The consortium was officially formed when the mathematical model and its accompanying online application were introduced to the first in-country teams. The model is an age-dependent susceptible-exposed-infected-recovered model, adapted from^55^ to reflect SARS-CoV-2 transmission/virological traits and the interventions being deployed in different countries—a detailed model description can be found in the online supplemental materials. A key focus was to build a SARS-CoV-2 specific model that could be seamlessly updated as new information became available and different sets of interventions were considered. A stand-alone Excel-based tool, the Cornell COVID-19 Caseload Calculator with Capacity and Ventilators model, formed the basis for the healthcare components of the CoMo Consortium model.^56^

10.1136/bmjgh-2020-003126.supp1 Supplementary data

From inception, the goal was to allow the user to define critical aspects of the model structure, model inputs, both in terms of parameter values and interventions considered, interface options and output reporting. This was key to the model being well accepted and easily adopted by so many different countries.

The consortium has grown organically since 16 March 2020 and now includes approximately 100 members, representing more than 30 countries (figure 1). Regular communication channels were established, and several developmental working groups were formed. These working groups are either country-specific, question-specific or technique-specific, with the latter including working groups for the mechanistic model, developing the web-based interface, exploring spatial formulations of the model and a hospital capacity simulation tool. Question-specific working groups include those centred around lockdown-release strategies, or screening and diagnostic strategies. The in-country experts are engaged in continuous communication with their respective policymakers, enabling the rapid adaptation of the CoMo Consortium model and other in-country models (where available) to address the fast-paced changes occurring during the course of the outbreak in each particular setting.

### CoMo Consortium—dissemination phase

**Figure 3:**
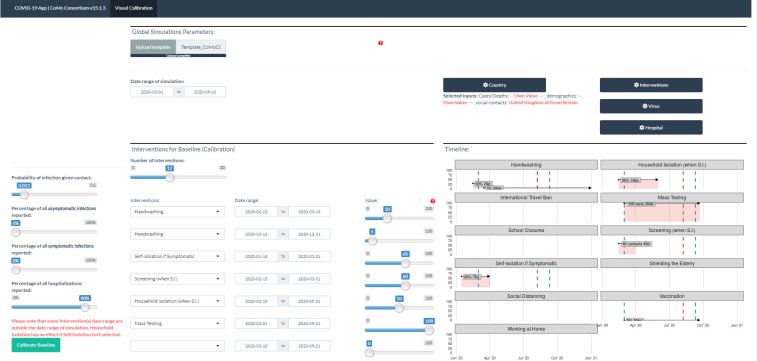
The CoMo model online application. Users can either upload a filled-in template or input all parameter values in the app directly. User can specify up to 30 intervention periods, defining the start and end dates, as well as the assumed coverage for each.

Following its initial development phase, the CoMo Consortium moved into the dissemination phase, once the model code had been sufficiently scrutinised and the interface—figure 3—had been redesigned to facilitate model calibration to data. A health economics working group was formed to identify the most appropriate use of available funds and inform future cost efficiency analyses. In-country working groups continued to work on their local COVID-19 situations while also contributing to and benefitting from the question-specific and technique-specific working groups. The full model code was made available to all members and a special online ‘code reading’ session was held. Members were then able to take the code and adapt it for their own local context. For example, the Brazil group has incorporated a modelling fitting algorithm while simplifying the hospital simulator submodel, while the Nigeria group has changed the age structure to reflect the age classes used by the Nigerian disease surveillance system.

An ongoing series of training sessions on the use of the model and the communication of results to policymaking partners was initiated by the consortium leader and then taken up by the members. Code version release notes are continually made available to all consortium members, highlighting any changes made and the reasons for those changes. A concerted effort was made to bridge the model code with the app code and test the performance of the model across code versions to ensure consistency.

At least one consultation was held between the development node and each country’s expert node to discuss parameter value assumptions and the contextual nature of some parameters, given how interventions are implemented locally. Some of these meetings instigated updates to the model structure and app interface customisation options.

### CoMo Consortium—use-cases

Three use-cases have emerged for the CoMo Consortium model and its accompanying web-based interface. First, in settings where there is already considerable translational capacity, the CoMo Consortium model is used by in-country modellers to crosscheck the models they have written, thus helping to refine their outputs and to maintain high accuracy and validity. Second, in settings where there is some coding expertise but less capacity to develop bespoke models, the CoMo Consortium’s primary code can be modified by in-country modellers to create country-specific models that are being used to assist policymakers’ decision-making. Third, in contexts where there is a desire among policymakers to use modelling to inform development of their strategies based on local parameter values, but limited capacity in terms of modelling or coding, the primary code is used via the CoMo Consortium model’s web-based interface.

Regardless of the use-case, CoMo operations are ruled by the flows of interaction depicted in figure 2. Clearly, the main catalysts are policy questions that shape the shared model that is adapted by each in-country expert team to generate country specific models. The in-country expert teams liaise with the technical experts to validate and debug their models and later proceed to communicate the model predictions to the policymakers in a comprehensible way. We cannot emphasise enough how critical the role played by the in-country experts is. It falls on them to engage with policymakers and explain to them the features and capabilities of the model, guide them in addressing appropriate modelling questions, and later package those questions alongside all relevant contextual factors to the technical team to ensure the model can represent the desired context appropriately. A few examples of the appreciation of the contextual nature of questions addressed by countries follow:

- In Syria, the main concern in the early stages of the epidemic was how the virus would spread in displaced populations and refugee camps. This could not be explored with the standard CoMo model, so a task team was put together to develop a bespoke refugee camp model.
- In New York, special attention was paid to the very fast upsurge in patients with respiratory distress in most city hospitals. This raised serious concerns on how countries with lower health capacity would fare, specifically to what concerns the number of available intensive care unit beds and ventilators. As a consequence, we invested quite some time developing the hospital submodel and have made it a central point of discussion with in-country modellers, as it is their role to assess the current and likely health capacity in their country and convey the message of how those limitations impact on the predicted epidemic mortality burden to their respective policymakers.
- In Afghanistan and Timor-Leste there was a lot of concern regarding the role of migrants in causing local outbreaks and seed local transmission. This led to the addition of a user defined parameter setting the number of daily imported infections.
- Interventions are not implemented in the same way everywhere, as country contextual idiosyncrasies critically affect the potential efficiency and practicability of any intervention.
  - Shielding of the elderly should be a major component of mortality burden reduction strategies in HICs, but are not feasible to employ in most LMICs, where people live in large familial households and it would not be possible for the elderly to isolate (and in some countries not culturally acceptable).
  - Self-isolation was a core component of initial containment strategies in Europe where it was quite straightforward and easily achievable for the individual person. In LMICs that is not that case, for the reasons highlighted above. Due to these issues with household structures, in Thailand, the ministry of health decided to isolate people fitting a clinical algorithm in governmental facilities, where they were kept for up to 14 days, or until they test negative for the virus. This type of testing centre was implemented early on in South Korea and Singapore to great effect.

The stage of engagement with local policymakers is quite heterogeneous across CoMo Consortium members (table 2). Members with existing channels of communication with Ministries of Health have gone through the process of providing feedback to their relevant authorities at a much faster rate. Some members of the academic community have needed to foster links with appropriate points of contact at Ministries of Health, specially appointed government entities, or COVID-19 taskforces, and establish appropriate channels of communication and gain the trust of policymakers. In some instances, it has been necessary for members to navigate a complex landscape, sometimes involving finding consensus among several different modelling groups offering advice to their government.

**Table 2:** CoMo Consortium member countries’ stages of engagement with policymakers

**Stage 1** Preliminary analyses and model calibration to explore optimal containment strategies	Argentina Bangladesh Brazil Cambodia Ecuador Ethiopia Nigeria Taiwan Sierra Leone Malaysia
**Stage 2** Have engaged with local Ministries of Health (MoH) or relevant policymakers and are in the process of analysing the different strategies under consideration	Philippines Portugal Myanmar Northwest Syria
**Stage 3** Have on at least one occasion presented CoMo Consortium model results to the local MoH or relevant policymakers	Afghanistan Cameroon Haiti Iran Lao PDR Tabasco Province, Mexico Queretaro State, Mexico Thailand Mozambique New York State, USA Orange County, California, USA Timor-Leste Nepal
**Stage 4** The local MoH has made policy decisions based on CoMo Consortium model predictions	Kyrgyzstan North Sumatra Province, Indonesia

CoMo, COVID-19 Modelling.

At the time of writing, 11 of the 22 countries from the WHO Regional Office for the Eastern Mediterranean (EMRO) have been using the CoMo Consortium model. Some are engaged in independent research using either the CoMo Consortium model (table 2), their own models, or both, while others are receiving active support from the WHO EMRO COVID-19 Modelling Support Group. The countries are distributed across all stages of policy engagement documented in table 2.

## Conclusion

Faced with the most significant pandemic in more than a century, we chose a participatory approach to create the international CoMo Consortium and develop a dynamic infectious disease model that addressed a global need. The key to the success of this participatory approach lies with the in-country expert node. The in-country experts include professionals with a wide range of expertise that play an essential iterative role, being both policy-facing in its dealings with policymakers and simultaneously delivering or facilitating reactive modelling that can feed back, in real time and based on the latest data, into the decision-making processes. Importantly, this continuous cooperation and feedback loop has been a valuable part of the process to facilitate collaboration and develop trust. A static online tool alone would not be sufficient to achieve this. The biggest strength of participatory approaches can ultimately be its largest limitation, as everything is reliant of the in-country expert being able to reach policymakers and/or gain access to the relevant data, perform data quality control, and liaise with the model development team to ensure the interventions being implemented in the field are well captured in the model.

Critically, our approach allows for tailoring of the model and online app to meet each country’s needs and facilitates translation of the analytical requirements into easily digestible outputs that can inform policy. The CoMo Consortium approach brings modelling to a broad range of people who will benefit from its participatory nature, through a combination of technology, training and effective communication.
