# Correction for modelling the COVID-19 pandemic in context: an international participatory approach

**DOI:** 10.1136/bmjgh-2020-003126corr1

**Published:** 2021-02-05

Aguas R, White L, Hupert N, *et al*. Modelling the COVID-19 pandemic in context: an international participatory approach. *BMJ Glob Health* 2020;5:e003126. doi: 10.1136/bmjgh-2020-003126.

This article has a correction. The group in South Africa has been using their own models and not the COVID-19 Modelling Consortium model. Their inclusion in the manuscript was an error.

