# Supplementary material for: Modelling the COVID-19 pandemic in context: an international participatory approach
Source: BMJ Glob Health. 2020 Dec 23;5(12):e003126. doi: 10.1136/bmjgh-2020-003126 (PMC7759758; doi:10.1136/bmjgh-2020-003126)
Supplement: Supplementary data [file bmjgh-2020-003126supp001.pdf]

BMJ Publishing Group Limited (BMJ) disclaims all liability and responsibility arising from any reliance placed on this supplemental material which has been supplied by the author(s)

Supplementary Materials

Full list of authors

Name	Affiliation
Lisa J White	Big Data Institute, Li Ka Shing Centre for Health Information and Discovery, Nuffield Department of Medicine, University of Oxford
Ricardo Águas	Mahidol-Oxford Tropical Medicine Research Unit, Faculty of Tropical Medicine, Mahidol University, Bangkok, Thailand.<br>Nuffield Department of Medicine, University of Oxford.
Nathaniel Hupert	Department of Medicine, Weill Cornell Medicine, and Cornell Institute for Disease and Disaster Preparedness, Cornell University, USA
Rima Shretta	Nuffield Department of Medicine,University of Oxford
Olivier Celhay	Mahidol-Oxford Tropical Medicine Research Unit, Faculty of Tropical Medicine, Mahidol University, Bangkok, Thailand
Ainura Moldokmatova	Big Data Institute, Li Ka Shing Centre for Health Information and Discovery, Nuffield Department of Medicine, University of Oxford
Proochista Ariana	Centre for Tropical Medicine and Global Health, Nuffield Department of Medicine, University of Oxford
Fatima Arifi	Florida International University
Ali Mirzazadeh	Department of Epidemiology and Biostatistics, Institute for Global Health Sciences, University of California San Francisco, San Francisco, CA, USA
Hamid Sharifi	HIV/STI Surveillance Research Center, and WHO Collaborating Center for HIV Surveillance, Institute for Futures Studies in Health, Kerman University of Medical Sciences, Kerman, Iran
Keyrellous Adib	Independent researcher (No affiliation)
Wirichada Pan-ngum	Mathematical And Economic MODelling Group, Mahidol-Oxford Tropical Medicine Research Unit, Faculty of Tropical Medicine, Mahidol University, Bangkok, Thailand
Penny Hancock	Big Data Institute, Li Ka Shing Centre for Health Information and Discovery, Nuffield Department of Medicine, University of Oxford
Roberto A. Kraenkel	Institute of Theoretical Physics - São Paulo State University (UNESP)
Renato Mendes Coutinho	Center for Mathematics, Computation and Cognition, Federal University of ABC, Brazil
Caroline Franco	Institute of Theoretical Physics - São Paulo State University (UNESP)
Sompob Saralamba	Mathematical And Economic MODelling Group, Mahidol-Oxford Tropical Medicine Research Unit, Faculty of Tropical Medicine, Mahidol University, Bangkok, Thailand
Nantasit Luangasanatip	Mathematical And Economic MODelling Group, Mahidol-Oxford Tropical Medicine Research Unit, Faculty of Tropical Medicine, Mahidol University, Bangkok, Thailand
Sheetal Prakash Silal	Modelling and Simulation Hub, Africa (MASHA), University of Cape Town, Cape Town, South Africa
Jared Norman	Modelling and Simulation Hub, Africa (MASHA), University of Cape Town, Cape Town, South Africa
Rachel Hounsell	Big Data Institute, Li Ka Shing Centre for Health Information and Discovery, Nuffield Department of Medicine, University of Oxford
Sai Thein Than Tun	1: Mathematical And Economic MODelling Group, Mahidol-Oxford Tropical Medicine Research Unit; 2: Nuffield Department of Medicine, University of Oxford
Yu Nandar Aung	London School of Economics
Bakare Emmanuel A.	Department of Mathematics, Federal University, Oye-Ekiti, Ekiti State, Nigeria.
Biniam Getachew	Stop Transmission of Polio Program (STOP) program, World Health Organization, Birnin Kebbi, Nigeria
Sandra Adele	Nuffield Department of Medicine, University of Oxford

Semeeh A. Omoleke	World Health Organization,Nigeria
Rashid U Zaman	Oxford Policy Management
Nicholas Letchford	Oxford Policy Management
Daniel M. Parker	Population Health and Disease Prevention; Department of Epidemiology; University of California-Irvine
Sunil Pokharel	Independent Researcher
Dipti Lata	Oxford Policy Management (OPM) Nepal
Siyu Chen	Nuffield Department of Medicine,University of Oxford
Shwe Sin Kyaw	Mahidol-Oxford Tropical Medicine Research Unit, Faculty of Tropical Medicine, Mahidol University
Mohammad Nadir Sahak	World Health Organization,Afghanistan
Inke N D Lubis	Department of Paediatrics, Faculty of Medicine, Universitas Sumatera Utara, Medan, Indonesia
Ivana Alona	Department of Community Medicine, Faculty of Medicine, Universitas Sumatera Utara, Medan, Indonesia
John Robert C. Medina	Department of Epidemiology and Biostatistics, College of Public Health, University of the Philippines Manila, Philippines
Chris Erwin G. Mercado	Independent Researcher
Sana Eybpoosh	Department of Epidemiology and Biostatistics, Research Centre for Emerging and Reemerging Infectious Diseases, Pasteur Institute of Iran, Tehran, Iran
Ibrahim Mamadu	World Health Organization,Nigeria
Manar Marzouk	Valid International Ltd
Nicole Feune de Colombi	Nuffield Department of Medicine, University of Oxford
Lorena Suárez-Idueta	Univesity College of London
Francisco Obando	PEAK Urban and Oxford Martin Informal Cities Programmes, COMPAS, School of Anthropology and Museum Ethnography, University of Oxford
Luzia Freitas	Nuffield Department of Medicine, University of Oxford
Michael G. Klein	Department of Marketing & Business Analytics, Lucas College and Graduate School of Business, San José State University, USA
David Scales	Department of Medicine, Weill Cornell Medicine, Cornell University, USA
Dooronbekova Aizhan	Public fund «Institution of social development»
Chynar Zhumalieva	Public fund «Institution of social development»
Aida Estebesova	USAID Mission in the Kyrgyz Republic
Aibek Mukambetov	Soros Foundation in the Kyrgyz Republic
Shamil Ibragimov	Soros Foundation in the Kyrgyz Republic
Aisuluu Kubatova	Public fund «Institution of social development»
Phetsavanh Chanthavialy	Institute of Research and Education Development, University of Health Sciences, Lao PDR
Amel H. Salim	Population Services International/Ethiopia
Sudhir Venkatesan	1: Real World Solutions, IQVIA, 2: Department of Medical Statistics, London School of Hygiene and Tropical Medicine
Sarin KC	Health Intervention and Technology Assessment Program (HITAP), Ministry of Public Health, Thailand
Priyanka Shrestha	Independent researcher (No affiliation)
Sayed Ataullah Saeedzai	Ministry of Public Health, Afghanistan
Jenny Hsieh	School of Public Health, National Taiwan University
Mick Soukavong	Department of Natural Sciences, Faculty of Medicine, University of Health Sciences, Laos
Yuki Yunanda	Department of Community Medicine, Faculty of Medicine, Universitas Sumatera Utara, Medan, Indonesia
Handoyo Harsono	COVID-19 Task Force for North Sumatera province, Indonesia
Mahnaz Hossain Fariba	Government of People's Republic of Bangladesh (ex. Nuffield Department of Medicine, University of Oxford)
Viviana Mabombo	Nuffield Department of Medicine, University of Oxford
Nicole Advani	Nuffield Department of Medicine, University of Oxford
Nusrat Jabin	Nuffield Department of Medicine, University of Oxford

BMJ Publishing Group Limited (BMJ) disclaims all liability and responsibility arising from any reliance placed on this supplemental material which has been supplied by the author(s)

Reshania Naidoo	Center for Tropical Medicine and Global Health, Nuffied Department of Medicine, University of Oxford
Parinda Wattanasri	Nuffield Department of Medicine, University of Oxford
Amen-Patrick Nwosu	Nuffield Department of Medicine, University of Oxford
Sopuruchukwu Obiesie	Center for Tropical Medicine and Global Health, Nuffied Department of Medicine, University of Oxford

Mathematical Model

The CoMo Consortium SARS-CoV-2 model is an age-structured SEIR model with infected compartments stratified by symptoms, severity and treatment seeking and access. The progression of individuals through the infection life cycle is represented by the diagram below.

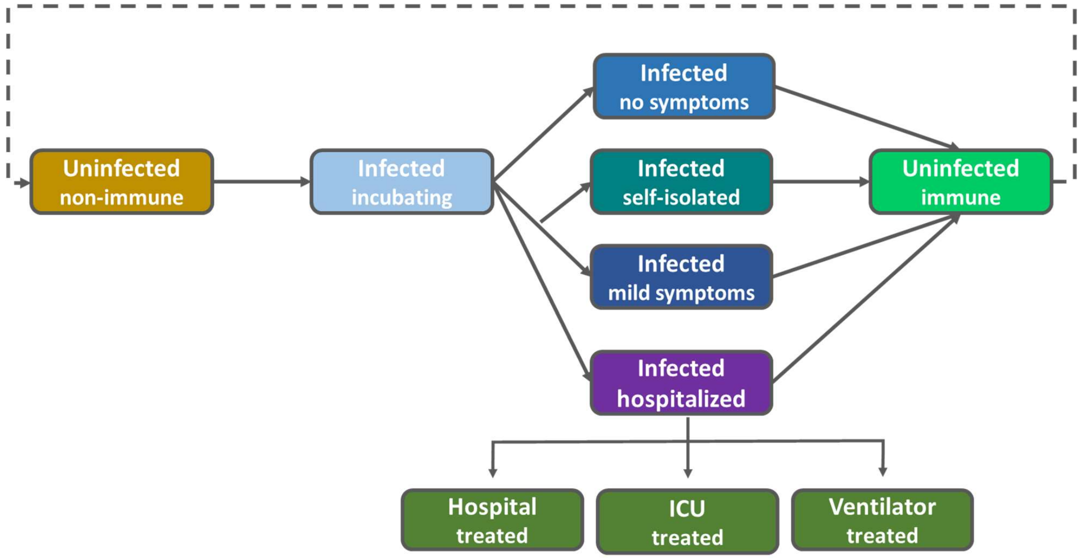

Figure S1: A diagram of the baseline model structure representing the unmitigated epidemic spread scenario

As individuals are infected, they transit through an incubation phase at the end of which they are fully infectious. At the end of this incubation period, individuals display very different symptomatology, with some never having any symptoms, while others require hospitalisation. If a person is hospitalised, the model tracks their health care requirements (surge care bed, ICU bed, ventilator). Disease-induced mortality rates are heavily dependent on how severe the infection outcome is and whether individuals can receive the appropriate treatment. The model assumes that those who recover from infection will become immune.

We allow for loss of immunity in the model but for the moment have set this parameter to be in the order of years. The equations for the basic model structure without non-pharmaceutical (NP) interventions follow

$$\frac{dS}{dt} = -S \circ \Lambda + \omega R + A \cdot S - \mu \cdot S + bP$$

$$\frac{dE}{dt} = S \circ \Lambda - \gamma E + A \cdot E - \mu \cdot E$$

$$\frac{dI}{dt} = \gamma\ (1 - p_{clin})(1 - p_{ihr}) \cdot E - \nu_I I + A \cdot I - \mu \cdot I$$

$$\frac{dC}{dt} = \gamma\ p_{clin}(1 - p_{ihr}) \cdot E - \nu_I C + A \cdot C - \mu \cdot C$$

$$\begin{aligned}\frac{dR}{dt} = {} & \nu_I(I + C) + A \cdot R - \omega R - \mu \cdot R + \left(1 - \delta_H\, p_{hf}\ \right)\nu_H \cdot H + \left(1 - \delta_{H_c}\, p_{hfr}\right)\nu_H \cdot H_c \\ & + \left(1 - \delta_U\, p_{hf}\ \right)\nu_U \cdot U + \left(1 - \delta_{U_c}\, p_{hf}\ \right)\nu_U \cdot U_c + \left(1 - \delta_{V_c}\, p_{hfr}\right)\nu_v \cdot U_{cv} \\ & + \left(1 - \delta_V\, p_{hfr}\right)\nu_V \cdot V + \left(1 - \delta_{V_c}\, p_{hfr}\right)\nu_V \cdot V_c\end{aligned}$$

$$\frac{dH}{dt} = p_{ihr}(1 - p_U)(1 - p_{K_H})\gamma E - \nu_H H + A \cdot H - \mu \cdot H$$

$$\frac{dH_c}{dt} = p_{ihr}(1 - p_U)p_{K_H}\gamma E - \nu_H H_c + A \cdot H_c - \mu \cdot H_c$$

$$\frac{dU}{dt} = p_{ihr}p_U\left(1 - p_{K_U}\right)(1 - p_V)\gamma E - \nu_U U + A \cdot U - \mu \cdot U$$

$$\frac{dU_c}{dt} = p_{ihr}p_U p_{K_U}(1 - p_V)\gamma E - \nu_U U_c + A \cdot U_c - \mu \cdot U_c$$

$$\frac{dU_{cv}}{dt} = p_{ihr}p_U p_{K_U}p_V\gamma E - \nu_V U_{cv} + A \cdot U_{cv} - \mu \cdot U_{cv}$$

$$\frac{dV}{dt} = p_{ihr}p_U\left(1 - p_{K_U}\right)\left(1 - p_{K_V}\right)p_V\gamma E - \nu_V V + A \cdot V - \mu \cdot V$$

$$\frac{dV_c}{dt} = p_{ihr}p_U\left(1 - p_{K_U}\right)p_{K_V}p_V\gamma E - \nu_V V_c + A \cdot V_c - \mu \cdot V_c$$

$$P = (S + E + I + C + R + H + H_c + U + U_C + U_{Cv} + V + V_c)$$

$$s = 1 + a\cos\left(2\pi\frac{\left(t - \left(\frac{365.25\phi}{12}\right) + t_{in}\right)}{365.25}\right)$$

$$W = W_{home} + W_{work} + W_{school} + W_{oth}$$

$$\Lambda = p\ s\ W\ \cdot\left(\frac{\rho E + I + C + \rho_s\ * (H + H_c + U + U_c + U_{cv} + V + V_c)}{P}\right)$$

$$A = \begin{pmatrix} \begin{pmatrix} -\alpha & 0 \\ \alpha & -\alpha \end{pmatrix} & \cdots & 0 \\ \vdots & \ddots \begin{pmatrix} -\alpha & 0 \\ \alpha & -\alpha \end{pmatrix} \ddots & \vdots \\ 0 & \cdots & \begin{pmatrix} -\alpha & 0 \\ \alpha & 0 \end{pmatrix} \end{pmatrix}$$

$$p_{K_H} = \left.\begin{matrix} 0\ for\ H < K_H \\ 1\ for\ H \geq K_H \end{matrix}\right\}$$

$$p_{K_U} = \left. \begin{matrix} 0\ for\ U < K_U \\ 1\ for\ U \geq K_U \end{matrix} \right\}$$

$$p_{K_V} = \left. \begin{matrix} 0\ for\ V < K_V \\ 1\ for\ V \geq K_V \end{matrix} \right\}$$

The model uses publicly available country-specific data to define the population structure[1], as a model input. A description of the variables used can be found in Table S1 and a list with all parameters included in the full model including non-pharmaceutical (NP) interventions is given in Table S2.

The model interface can be found here: https://comomodel.net/. The model interface uses publicly available country-specific data on cases and mortality for COVID-19[1] for visual calibration of model parameters to user-selected baseline scenarios. More elaborate calibration methods are under consideration but are not considered to be a priority since the system is unidentifiable and therefore calibration will be entirely dependent on user-selected baseline scenarios.

Non-pharmaceutical interventions

A series of non-pharmaceutical interventions were included in the model; these can be switched on for specific periods of time, thus building a bespoke intervention package.

Self-Isolation if Symptomatic

This is the practice of individuals with either a confirmed case of Covid-19 or with Covid-19 symptoms isolating themselves at home for a period of 7 days. The parameters governing this intervention are:

- Start Date: the start date of the protocol
- Duration: the duration of the protocol
- Coverage: the percentage of the population who will be able to self-isolate if they have symptoms or are a confirmed case
- Adherence: the percentage of the designated isolation period that self-isolated individuals adhere to the intervention

Screening

This is a form of contact-tracing. Given enough testing capacity, it reflects how suspected contacts of confirmed cases are tested using a SARS-CoV-2 virological test. All individuals who test positive are then requested to self-isolate

- Start Date: the start date of additional screening
- Test Sensitivity: Probability that an infected person will test positive when screened
- Suspected Contacts: number of people screened per reported case
- Overdispersion: informs the probability of finding an infected person that is a known contact of a reported case, relative to random sampling (overdispersion = 1)

- Duration: duration of this additional protocol

Physical Distancing

Also known as physical distancing, this refers to the measures taken to prevent the spread of a contagious disease by maintaining a specific physical distance between individuals and reducing the number of times individuals come into close contact with each other. The parameters governing this intervention are:

- Start Date: the start date of the protocol
- Duration: the duration of the protocol
- Coverage: the percentage of the population who reduce their societal contacts (excluding those at home, work and school)
- Adherence: the percentage of the time that those practicing physical distancing adhere to physical distancing measures

Handwashing

This indicates improvements in personal hygiene and reduction in risk behaviours (touching the face, nose or mouth), including the adoption of personal protective equipment such as masks. The parameters governing this intervention are:

- Start Date: the start date of the protocol
- Duration: the duration of the protocol
- Efficacy: the effectiveness of personal hygiene measures in reducing the risk of infection per contact

Working at Home

This indicates the effect of having workers working from home. The parameters governing this intervention are:

- Start Date: the start date of the protocol
- Duration: the duration of the protocol
- Efficacy: the percent reduction in work related contacts
- Home contacts inflation: the percent increase in the numbers of home contacts due to increased number of hours spent at home

School Closures

This indicates school closures and assumes that all schools in a country close at the same time. The parameters governing this intervention are:

- Start Date: the start date of the protocol
- Duration: the duration of the protocol
- Efficacy: defined as the percent reduction in contacts between school children when schools are closed

- Home contacts inflation: the percent increase in the numbers of home contacts due to increased numbers of hours spent at home

Shielding the Elderly

This intervention is designed to isolate a proportion of the elderly population and reduce their overall contacts. The parameters governing this intervention are:

- Start Date: the start date of the protocol
- Duration: the duration of the protocol
- Coverage: the percentage of the elderly population who are shielded
- Efficacy: defined as the percent reduction in overall contacts of the shielded elderly population
- Minimum age for elderly cocoon: the minimum age cut-off defining which people should protect themselves

Travel Ban

This refers to a ban on international travel. The parameters governing this intervention are:

- Start Date: the start date of the protocol
- Duration: the duration of the protocol
- Efficacy: the percent reduction in imported cases per day

Voluntary home quarantine

This indicates how many people will voluntarily quarantine themselves at home for a specified number of days if a person they live with tests positive for Covid-19. The parameters governing this intervention are:

- Start Date: the start date of the protocol
- Duration: the duration of the protocol
- Days in quarantine for an average person
- Coverage: The percentage of people voluntarily quarantining themselves given they live with a known infectious case
- Rate: Speed at which people decide to quarantine themselves if they live with a known infectious case
- Percent decrease in the number of other contacts when voluntarily quarantining: refers to decreased mean numbers of contacts outside of the home while quarantining
- Percent increase in the number of contacts at home when voluntarily quarantining: refers to increased numbers of home contacts due to increased time spent at home while quarantining

Lockdown

This refers to an emergency protocol that is categorised into three levels based on the efficacy or coverage of the various non-pharmaceutical interventions. The parameters governing this intervention are:

- Low-level lockdown (self-isolation 50%, physical distancing 25%, cocoon 95%, hand-hygiene 5%)
    - Start Date: the start date of mid-level lockdown protocols
    - Duration: the duration of mid-level lockdown protocols
- Mid-level lockdown (self-isolation 50%, physical distancing 95%, school closure 85%, travel ban 95%, voluntary home quarantine 5%, working from home 50%, cocoon 95%, hand-hygiene 5%)
    - Start Date: the start date of low-level lockdown protocols
    - Duration: the duration of low-level lockdown protocols
- High-level lockdown (self-isolation 95%, physical distancing 35%, school closure 85%, voluntary home quarantine 90%, working from home 75%, cocoon 95%, hand-hygiene 7.5%)
    - Start Date: the start date of high-level lockdown protocols
    - Duration: the duration of high-level lockdown protocols

Table S1. Description of the model variables.

Symbol	Definition
S	Susceptible
E	Infected and incubating
I	Infectious and asymptomatic following incubation
C	Infectious and mildly symptomatic following incubation
R	Recovered and immune
H	Severe infection: hospitalised
H_c	Severe infection: not hospitalised due to lack of capacity
U	Severe infection: hospitalised in ICU
U_c	Severe infection: hospitalised and requiring ICU but placed in surge ward
U_{cv}	Severe infection: hospitalised and requiring ventilator but placed in surge ward
V	Severe infection: hospitalised in ICU and on a ventilator
V_c	Severe infection: hospitalised in ICU requiring a ventilator but not on one

Table S2. A list of the default parameter values used. These are subject to change when the model is applied to a new setting and/or with new incoming information. We have provided references to demonstrate that the default values lie within plausible ranges. † Country-specific value; ‡Assumed value (no reference found).

Symbol	Definition	Value	Unit	Source
**Demographics**				
W_{home}	Country-specific age-dependent contact matrix describing the number of potentially infectious contacts at home per person per day	†	day^{-1}	[2]
W_{work}	Country-specific age-dependent contact matrix describing the number of potentially infectious contacts at work per person per day	†	day^{-1}	[2]
W_{school}	Country-specific age-dependent contact matrix describing the number of potentially infectious contacts at school per person per day	†	day^{-1}	[2]
W_{other}	Country-specific age-dependent contact matrix describing the number of potentially infectious societal contacts per person per day	†	day^{-1}	[2]
μ	1/Age-dependent non-Covid-19-related death rate	†	days	[3]
b	1/ Age-dependent fertility rate	†	days	[3]
α	Ageing rate between age categories	0.2	$year^{-1}$	
**Natural history of infection**				
p	Probability of infection given a single contact	†	NA	[4]
γ	1/duration of incubation period	3.5	days	[5-7]
ρ	Relative infectiousness of incubating phase	0.1	NA	‡
p_{clin}	Proportion of all infections that ever develop symptoms	0.55	NA	[8-10]
ν_I	1/duration of infectious phase post incubation	4.5	days	[5]
ρ_s	Relative proportion of contacts for hospitalised patients	0.15	NA	‡
ω	1/duration of immunity	150	years	‡
**Seasonality**				
a	Relative variation in viral transmissibility throughout the year (+- a proportion)	†	NA	-
ϕ	Month of peak in transmissibility	†	NA	-
**Patient outcomes**				
p_{ihr}	Probability of an infection being severe (requiring hospitalisation) by age	†	NA	[3 11-14]
p_{hfr}	Probability of a severe/hospitalised infection being fatal by age	†	NA	[3 11 12 14 15]
ν_H	1/Duration of hospitalised infection	†	days	[16]
ν_U	1/Duration of ICU infection	†	days	[17 18]

$\boldsymbol{\nu_V}$	1/Duration of ventilated infection	†	days	5 6 15
$\boldsymbol{\delta_H}$	Maximum probability of death for a hospitalised infection	0.35		3 16
$\boldsymbol{\delta_{H_c}}$	Maximum probability of death for an infection requiring hospitalisation that did not receive appropriate treatment	0.45	NA	19
$\boldsymbol{\delta_U}$	Maximum probability of death for a hospitalised infection requiring ICU admission	0.55	NA	19 20
$\boldsymbol{\delta_{U_c}}$	Maximum probability of death for a hospitalised infection that would require ICU admission but was not admitted to the ICU	0.8	NA	19
$\boldsymbol{\delta_V}$	Maximum probability of death for a hospitalised infection requiring a ventilator	0.8	NA	19
$\boldsymbol{\delta_{V_c}}$	Maximum probability of death for a hospitalised infection that would require a ventilator but did not get one	0.95	NA	21
$\boldsymbol{p_U}$	Probability of an infected patient needing ICU	0.5	NA	13 21
$\boldsymbol{p_V}$	Probability of an infected patient needing ICU and a ventilator	0.75	NA	21
$\boldsymbol{K_H}$	Standard hospital bed capacity	†	NA	-
$\boldsymbol{K_U}$	ICU bed capacity	†	NA	-
$\boldsymbol{K_V}$	Ventilator capacity	†	NA	-

References

1. World Population Prospects 2019: United Nations; 2020 [Available from: https://population.un.org/wpp/Download/Standard/Population/.
2. Prem K, Cook AR, Jit M. Projecting social contact matrices in 152 countries using contact surveys and demographic data. *PLoS Comput Biol* 2017;13(9):e1005697. doi: 10.1371/journal.pcbi.1005697 [published Online First: 2017/09/12]
3. COVID-19: Data 2020 [Available from: https://www1.nyc.gov/site/doh/covid/covid-19-data.page.
4. Otto MA. COVID-19 update: Transmission 5% or less among close contacts 2020 [Available from: https://www.the-hospitalist.org/hospitalist/article/218769/coronavirus-updates/covid-19-update-transmission-5-or-less-among-close.
5. Linton NM, Kobayashi T, Yang Y, et al. Incubation Period and Other Epidemiological Characteristics of 2019 Novel Coronavirus Infections with Right Truncation: A Statistical Analysis of Publicly Available Case Data. *J Clin Med* 2020;9(2) doi: 10.3390/jcm9020538 [published Online First: 2020/02/17]
6. Khalili M, Karamouzian M, Nasiri N, et al. Epidemiological Characteristics of COVID-19; a Systematic Review and Meta-Analysis. *Plos One* 2020;Submitted

7. Bi Q, Wu Y, Mei S, et al. Epidemiology and Transmission of COVID-19 in Shenzhen China: Analysis of 391 cases and 1,286 of their close contacts. *medRxiv* 2020 doi: 10.1101/2020.03.03.20028423

8. Mizumoto K, Kagaya K, Zarebski A, et al. Estimating the Asymptomatic Proportion of 2019 Novel Coronavirus onboard the Princess Cruises Ship, 2020. *medRxiv* 2020:2020.02.20.20025866. doi: 10.1101/2020.02.20.20025866

9. Day M. Covid-19: four fifths of cases are asymptomatic, China figures indicate. *BMJ* 2020;369:m1375. doi: 10.1136/bmj.m1375 [published Online First: 2020/04/02]

10. Coronavirus disease 2019 (COVID-19) pandemic: increased transmission in the EU/EEA and the UK – seventh update [Available from: https://www.ecdc.europa.eu/sites/default/files/documents/RRA-seventh-update-Outbreak-of-coronavirus-disease-COVID-19.pdf.

11. Vital Surveillances: The Epidemiological Characteristics of an Outbreak of 2019 Novel Coronavirus Diseases (COVID-19) — China, 2020 [Available from: http://weekly.chinacdc.cn/en/article/id/e53946e2-c6c4-41e9-9a9b-fea8db1a8f51.

12. Hauser A, Counotte MJ, Margossian CC, et al. Estimation of SARS-CoV-2 mortality during the early stages of an epidemic: a modelling study in Hubei, China and northern Italy. *medRxiv* 2020:2020.03.04.20031104. doi: 10.1101/2020.03.04.20031104

13. Verity R, Okell LC, Dorigatti I, et al. Estimates of the severity of coronavirus disease 2019: a model-based analysis. *Lancet Infect Dis* 2020 doi: 10.1016/s1473-3099(20)30243-7 [published Online First: 2020/04/03]

14. Coronavirus: summary of persons being monitored, persons under investigation, and cases [Available from: https://floridadisaster.org/globalassets/covid19/dailies/covid-19-data---daily-report-2020-04-25-0956.pdf.

15. ICNARC report on COVID-19 in critical care [Available from: icnarc.org.

16. Zhou F, Yu T, Du R, et al. Clinical course and risk factors for mortality of adult inpatients with COVID-19 in Wuhan, China: a retrospective cohort study. *Lancet* 2020;395:1054-62. doi: 10.1016/S0140-6736(20)30566-3 [published Online First: 2020/03/11]

17. Wang Z, Ji JS, Liu Y, et al. Survival analysis of hospital length of stay of novel coronavirus (COVID-19) pneumonia patients in Sichuan, China. *medRxiv* 2020:2020.04.07.20057299. doi: 10.1101/2020.04.07.20057299

18. Huang C, Wang Y, Li X, et al. Clinical features of patients infected with 2019 novel coronavirus in Wuhan, China. *Lancet* 2020;395(10223):497-506. doi: 10.1016/s0140-6736(20)30183-5 [published Online First: 2020/01/28]

19. Petrilli CM, Jones SA, Yang J, et al. Factors associated with hospitalization and critical illness among 4,103 patients with COVID-19 disease in New York City. *medRxiv* 2020:2020.04.08.20057794. doi: 10.1101/2020.04.08.20057794

20. Lewnard JA, Liu VX, Jackson ML, et al. Incidence, clinical outcomes, and transmission dynamics of hospitalized 2019 coronavirus disease among 9,596,321 individuals residing in California and Washington, United States: a prospective cohort study. *medRxiv* 2020:2020.04.12.20062943. doi: 10.1101/2020.04.12.20062943

21. Richardson S, Hirsch JS, Narasimhan M, et al. Presenting Characteristics, Comorbidities, and Outcomes Among 5700 Patients Hospitalized With COVID-19 in the New York City Area. *JAMA* 2020 doi: 10.1001/jama.2020.6775 [published Online First: 2020/04/22]
